# Evaluation of the use of a handheld prescribing card in supporting Foundation year one doctors in End of Life (EOL) prescribing

**DOI:** 10.15694/mep.2019.000157.1

**Published:** 2019-07-24

**Authors:** Natalie Walker, Cathy Sherratt

**Affiliations:** 1 Royal Bolton Hospital; 2 Edge Hill University

**Keywords:** palliative care, junior doctors, education

## Abstract

This article was migrated. The article was marked as recommended.

Background

Literature reports disquiet in junior doctors’ abilities in palliative care prescribing; including distress and low confidence ( Charlton and Smith, 2000). We confirmed similar findings following local research thus identifying a development need. This led to the design and implementation of a hand-held prescribing card.

Objective

Usefulness of the prescribing card in supporting foundation year one doctors was evaluated. We hypothesised this intervention would help improve End of Life (EOL) care.

Methods

A mixed methods approach was employed using a specially designed questionnaire, distributed to 39 foundation year one doctors (doctors in their first year of practice after graduating from medical school). Focused questions were on utilisation, levels of prescribing confidence and exploring further interventions that might help, as well as feedback on card design.

Results

25 doctors completed questionnaires; a response rate 86%. Almost half routinely used the card. 40% were not yet prescribing for EOL situations at the time of the study because of their specific job rotation (e.g. ophthalmology). The commonest motivator was accessibility. All doctors reported increased confidence in prescribing and approximately three quarters said it enhanced practice. “Usefulness” was the commonest free-response descriptor. Feelings included it being a good reference and preventing errors. A development idea included an electronic version.

Conclusion

Results highlight that a simple hand-held prescribing card is useful. To our knowledge, this is the first UK study of its kind employing an educational intervention in palliative care in a hospital setting. It is important to implement this educational intervention early to support doctors.

## Introduction

**‘Palliative care is the right of every patient and its provision the responsibility of every doctor’** ( Doyle, 1996 cited in Charlton & Smith, 2000 p. 27).

Worldwide there is considerable evidence that foundation doctors feel unprepared to care for dying patients ( Gibbins *et al*., 2011). Indeed, Field and Howell (1998) surveyed junior doctors and found self-reported adverse feelings of vulnerability and distress. This is of concern considering junior doctors are on the front line looking after this cohort of patients. Lack of consistency in undergraduate palliative care teaching may help to explain. Certainly, evidence suggests it to be fragmented, ad-hoc and time limited ( Price and Schofield, 2015).

After careful reflection on the above, we explored challenges amongst foundation doctors locally, working in End of Life (EOL) care and considered educational interventions that might help them improve. This was in line with national end of life directives which stress the role of educational development in EOL care ( Royal College of Physicians, 2016).

A survey was designed and completed with questions addressing confidence in different areas of EOL prescribing and identifying perceived teaching needs (Supplemetary file 2). The second stage involved focus group interviews to further explore confidence. In summary, results showed low levels of confidence in EOL prescribing mirroring previous research findings. Subsequently a handheld card (Supplementary file 1) was developed, launched into clinical practice and evaluated.

It is meaningful to conduct this work as there is a paucity of evidence on educational research in this area ( Bowden *et al*., 2013). Evolving this domain is crucial for supporting our doctors early in their career.

## Literature review

Key findings from this field of research identifies newly qualified doctors feel unprepared and unconfident to care for patients at the EOL ( Gibbins *et al*., 2011; Price and Schofield, 2015). This might be simply explained by lack of training and there is a wealth of literature recognising insufficient training in palliative care in medical School curricula ( Lloyd-Williams and Macleod, 2004; Field and Howell, 1998; Charlton and Smith, 2000).

It can perhaps be explained by the fact that Palliative care itself is a relatively new speciality ( JRCPTB, 2016) and therefore its only recently that all medical schools include mandatory modular teaching on palliative care ( Lloyd-Williams and MacLeod, 2004, Walker *et al*., 2014). Also, palliative care is a small field so even the most enthusiastic teachers are frequently busy, and time is limited. Improvements have been made and by 2008, the association for palliative medicine published a curriculum which was recommended for undergraduate teaching ( Bowden *et al*., 2013). Before this, palliative care had not been a priority topic in medical schools.

It is not surprising then that newly-qualified doctors report gaps in knowledge, skills and behaviours when it comes to EOL care provision. Price and Schofield (2015) further point out that most of skills in palliative care are developed by qualified doctors, often by experiential learning, as again formal integrated EOL teaching is at a premium. One study of doctors, in 2003, reported their education in palliative care as a junior doctor was negligible ( Charlton and Currie, 2008).

There is little data available in the UK that has focused on implementing learning strategies amongst foundation doctors and developing specific post-graduate training programmes. Most previously available data is based around Medical School education. So, we really are at the tip of the iceberg when it comes to secondary care post-graduate palliative care training. However, education is clearly at the heart of palliative care (The National Association of Palliative Care Educators, NAPCE, 2016) and this project has worked towards addressing some of these needs in a local population of foundation doctors by offering an educational intervention.

Globally, there are two published Canadian papers demonstrating the benefit of focused educational projects to improve EOL care provision for doctors. Both showed written guidance was useful. Mikhael *et al*., (2008) provided a pocket card for pain and symptom control alongside blended teaching. Critchley *et al*., (2002) also designed a pocket card to help with pain and symptom control prescribing with positive results. We hoped our work would echo these findings.

## Methods

This study gained ethical approval from the research governance team within Bolton NHS Foundation Trust and was registered at Edge Hill University. Project funding was granted for the development of the handheld card by the faculty of Medical Education at Bolton NHS Foundation Trust. The nature of the study was explained to the participants in an information sheet, distributed one week prior to data collection. For data collection an anonymous specially-designed questionnaire (Supplementary file 2) was distributed to the population group of 39-foundation year one doctors (known in some countries as ‘interns’) from our local hospital; a convenience sample. Inclusion criteria were all the doctors attending foundation year one professional development teaching on the day of data collection. The only exclusion criteria were refusal to participate in the study. It was expected that the junior doctors completed the questionnaires over a period of 15 mins.

Initial questions covered demographic data with simple dichotomous responses. The next questions used multiple-choice design to investigate how much the card was utilised as well as exploring motivational factors and barriers. The following section assessed confidence in aspects of EOL care prescribing; a one directional anchor scale was used to measure responses. The next stage of the questionnaire focused on satisfaction of the educational intervention using a Likert style response. Views on other interventions of potential use were then asked about before a final question allowing opportunity to give feedback on card design.

## Data Analysis

Quantitative data was analysed using descriptive statistics as the sample size was small. The total number of participants on the day of the survey was 29 out of a possible 39 (74%). 25 doctors out of 29 completed questionnaires, a response rate of 86%. 20 questionnaires were incomplete although none unfilled to a significant degree. All questionnaires were analysed. The majority of participants (80%, n=20) were between 20-25 years of age with 20% (n=5) being over age 25. There were almost equal numbers of males and females which matches the population of newly-qualified doctors in this region.

At the time of the study, 48% (12) of the sample of foundation year one doctors reported to be routinely using the card. Males favoured its use overall more frequently than females and the more mature doctors used the card more; gender and age therefore both appear to be influencing factors. Simply forgetting to bring the card to work on occasion was the biggest inhibiting factor preventing its use; almost half of the doctors surveyed admitted to this.

**Figure 1.**
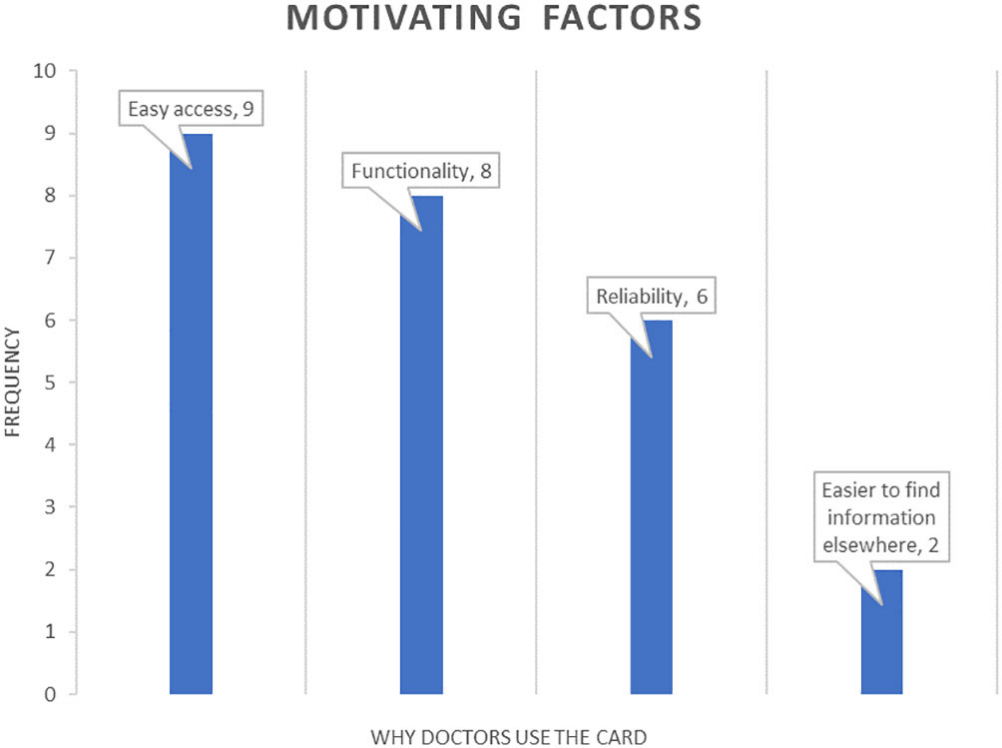
Motivating factors

The maximum response for motivating factors for using the card was easy access, with 25% of participants thinking this important. Just under a 1/3 believed its functionality was an important driver and almost a quarter (24%) reported reliability ( Figure 1).

**Figure 2.**
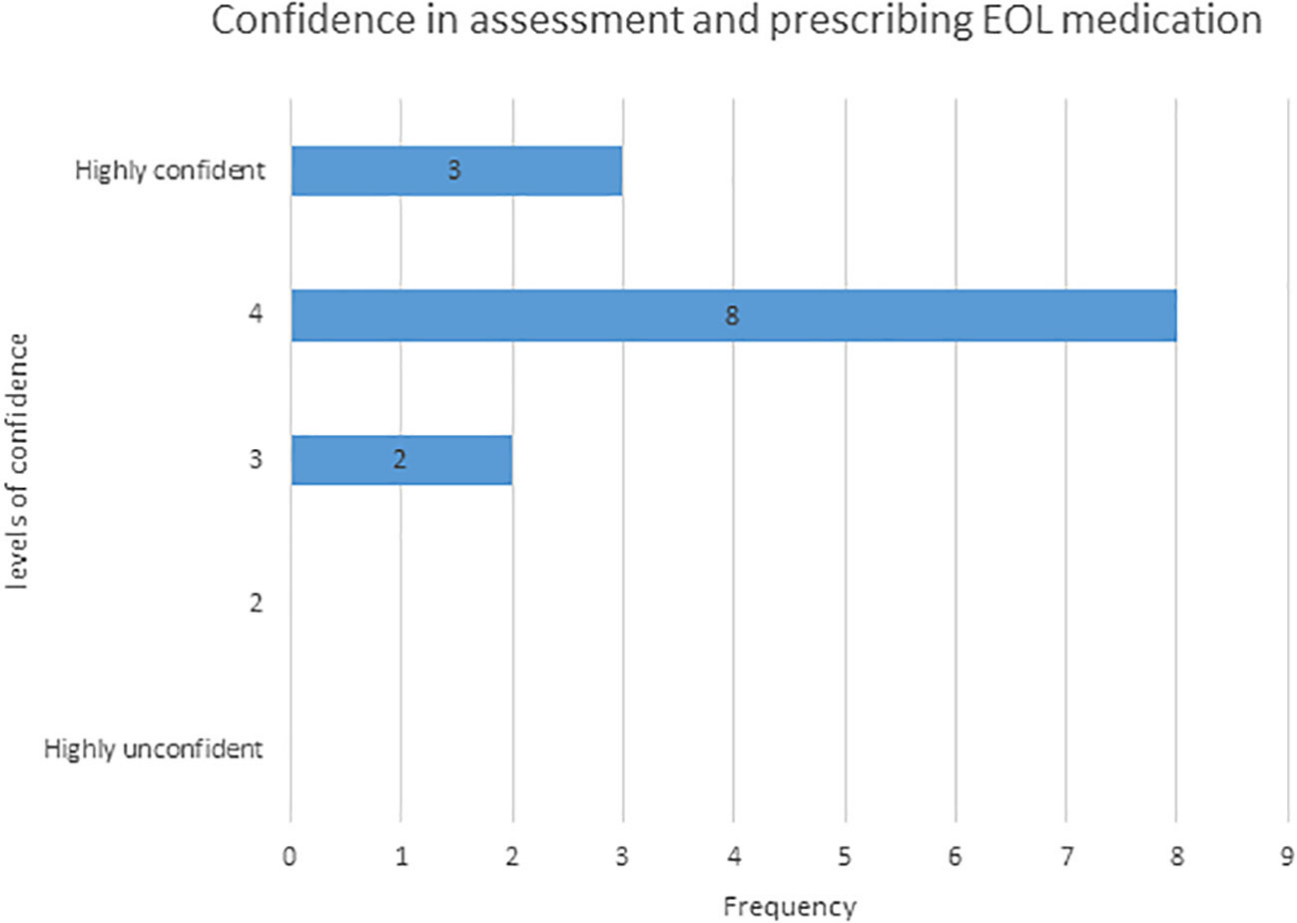
Confidence in EOL prescribing

We noticed that there was a positive association with the introduction of the card and increased confidence in all three domains of prescribing. All respondents reported increased confidence in the assessment and prescribing of EOL medications with the use of the hand-held card; almost a quarter of respondents (23%, 4) perceived themselves to be highly confident. By using the card, all responders also reported increased confidence in drug conversion and 40% felt more confident in titrating analgesia with the use of the card. ( Figure 2)

74% of participants were very satisfied or satisfied that the hand-held card had enhanced their clinical practice. The commonest recurring feeling towards the card was that of its **“usefulness”. “Accessibility”** was also commonly described. Further emergent themes reported included it being a **“good reference”** and **“preventing errors”**.

It is important to consider ideas for improvements and this included **“developing an electronic version”, “practical sessions on its use”, “making it smaller” and “larger copies for the ward walls.”**

## Discussion

To our knowledge this is the first service evaluation study concerning education support for foundation doctors providing EOL care in the UK.

25 out of the 29 possible doctors completed the questionnaires within 15 minutes, demonstrating that the questionnaire was simple to use. The final response rate was 86%. However, 20 questionnaires were not fully completed for unknown reasons. It is not known whether this was due to a reluctance to complete, poor comprehension, a failure of the visual layout of the questionnaire or possibly a lack of time. All questionnaires were analysed for data capture given the numbers overall were small.

Approximately ¾ of the doctors reported that they had their own copy of the card. It is not clear why ¼ did not have their own copy, possibly attributable to the method of initial distribution. On the day the cards were given out, they were left on a table for collection. In retrospect they should have been handed directly to the individuals to ensure that every junior doctor was fully supported in EOL prescribing.

Although this is a small case study, the level of utilisation of the hand-held card in our study is high. Almost 50% of participants reported to be using the card at the time of the survey with more than a third using it monthly and almost 10% on a weekly basis. Some participants commented that the card was not relevant to them at the time of survey due to their current job rotation e.g. sexual health, but that they would use it in another job. This reflects the variety of possible placements experienced by our newly-qualified doctors, on a rotational basis.

There were differences shown between the genders with males favouring using the card overall on a more frequent than the females and age did make a difference also with the mature students favouring its use. It was interesting to note that 80% of the more mature students (>25) used the cards in totality whereas 70% of the 20-25-year olds used the cards. A simple recall can be more difficult for some than others and might be an explanation. A visual prompt may be more desirable to some.

More than a third of responders said accessibility of the card motivated them to use it. Charlton and Currie (2008) previously reported lack of access as one barrier to palliative care training; the hand-held card is therefore one solution. Indeed 25% of participants selected reliability as their motivational factor for using the card. This correlates with literature suggesting that junior doctors require specific education which can support everyday work ( RCP, 2016).

In terms of inhibiting factors, our results reinforced that simply forgetting to bring the card to work stopped almost half of the doctor using the card (46%). It could be postulated that the results of the questionnaire might have been quite different with a larger number of doctors using the card and a follow-up evaluation is therefore recommended for future cohorts.

This next stage of the questionnaire was extremely important because in line with previous research, we recognise that levels of confidence in EOL care for junior doctors is low ( Gibbins *et al*., 2010; Hull, 1991; Bowden *et al*., 2012). These questions were not answered by all active participants and it is not clear whether the style of question was difficult to answer, if the participants chose not to answer it due to “fear” of admitting low levels of confidence ( Sturman *et al*., 2017), or if the layout of the questionnaire meant that some people simply overlooked the questions. Nonetheless, from those that answered the questions, the results showed subjective increased levels overall of confidence in all three areas surveyed: - assessment and prescribing in EOL care, drug conversions and drug titrations.

The highest level of confidence in assessment and prescribing for EOL care accounted for just over 1/5 of all responders which is a significant improvement following the introduction of the card. Almost 2/3 reported high self-perceived improvements in confidence. Improvement in confidence for drug conversions with the use of the hand-held card was high and 50% of responses reported a high level of confidence. We believe this reflects the fact that the card allows for generic conversions and gives doctors the ability to use the card in everyday clinical situations suggesting its flexibility and usability on the wards. The last domain concentrates on drug titration and the use of the card can provide additional support to this end, not previously covered. Importantly, an improvement was reported to some degree from all participants, even those who did not report a big increase in confidence. However, 42% of doctors reported a big improvement in their confidence levels and 35 % moderate, again reflecting the fact that the card is specifically applicable to daily use. It should also be remembered that some participants may have felt subject to reporting bias and it possible that there could have been both under and over reporting of confidence across the domains.

We found ¾ of responders were satisfied that the intervention enhanced clinical practice with 32% being very satisfied. None of our participants reported that the card negatively impact clinical practice. Although the study has not objectively measured improvement, self-perceived enhancement with this simple educational tool is clear to see in the results. As it was a self-reported study it is possible some doctors might over and under report confidence, so it would be useful to repeat the study with another cohort of doctors to look at overall trends and draw comparisons. Nevertheless, the self-reported enhancement with the utilisation of the card provides hope for the future foundation doctors and the hospital is keen to roll out the project. To make this project more robust, it may be sensible, to introduce an objective measurement to analyse this education intervention in more detail. Approximately 1/5 of responders were undecided about the hand-held card. Some of this group had commented that they had not yet had much opportunity to use due to their current placements. Had the project been completed later, for example, at the end of foundation year one, it is possible that more participants might have reported a higher level of satisfaction. This is, however, purely speculative and could suggest a need for a further phase of the study.

The next part of the survey asked doctors to communicate interventions that they felt might further improve their education over and above the hand-held card. Almost 50% suggested specific sessions with the palliative care team whereas a 1/3 said they would welcome a taster day within palliative care and 20% of participants though that peer teaching sessions would be helpful. No one method used in isolation is satisfactory for all individuals; flexibility of approach is crucial ( Gibbins *et al*., 2010).

The final section of the evaluation focused on feelings towards the card asking for opinions. A common theme was that the card was “useful” inferring that use of the card had been a positive experience. Similarly, other positive free responses recorded were that the card was “accessible”, “great”, “good idea”, “good format” and “easy to use”. Feedback also suggested that the card “prevented error” and was a “good reference” with “good content”. Potential ideas for the future included developing an electronic version for use on mobile phones.

Overall, results are encouraging and confirms our hypothesis that the card is a useful educational intervention. This is in line with world-wide literature, suggesting credibility and transferability. Furthermore, there is a potential greater reach for the project; one doctor suggested that cards be redistributed to other grades of doctors for enhanced effect.

Despite the overall positive feedback, one doctor reported that the card was intimidating as it was scary to prescribe these drugs. This is quite an emotional response and important to capture. It is important to recognise this specific feedback as it reflects the fact that further training and intervention is still required. This doctor appears to be quite vulnerable and lacking in confidence, and may therefore be at risk of making a mistake. Such feedback might not have been addressed if a free text response question had not been integrally incorporated into the questionnaire. There was surprisingly very little specific feedback on the appearance of the card itself other than two doctors comments; one felt that card was too big, and another believed that the edges of the card were too sharp.

## Conclusion

Self-reported usefulness of the hand-held card to support foundation doctors in EOL care prescribing in this study was high with almost half of the participating doctors using the card in every day clinical practice. Accessibility, functionality and reliability were the most common motivating reasons for using the card whilst simply forgetting to bring the card to work represented the biggest inhibiting factor. All doctors reported increased confidence in all three domains of clinical care surveyed and 75% of doctors were either satisfied or very satisfied that the use of this intervention enhanced their clinical practice. Likeable factors of the card included it being a good reference and preventing errors. Ideas for improvements were offered and these included additional teaching sessions alongside owning the card and a potential electronic version for use on mobile technology.

Questionnaires were easy to administer, and the survey received a high response rate of 86%. In line with previous literature, this service evaluation study confirms the important role of educational intervention in palliative care. Our results have shown that it is useful, easily implementable, cheap, portable, reliable and accessible. To our knowledge, this is the first UK study of its kind employing an intervention in palliative care in a hospital setting. It is important to implement this intervention early to support junior doctors early in their careers. Moving forwards, we would propose an ultimate vision of a standardised schedule of palliative care training from undergraduate training through to postgraduate working in a drive to ensure quality and safety and streamline the provision of EOL care.

## Glossary of terms

EOL - End of Life care

Foundation year one doctors - first year doctor after graduation from UK medical school

## Take Home Messages

• A hand-held prescribing prompt card is considered “useful” in enhancing clinical practice for junior doctors
• Using a hand-held prescribing card improves confidence in all areas of EOL care prescribing
• It is easily implementable and cheap
• Portability allows easy accessibility and reduces errors
• It is important to introduce this intervention early in a junior doctor’s career

## Notes On Contributors

Dr Natalie Walker, Consultant Physician and Associate Foundation Programme Director - Bolton NHS Foundation Trust - BSc(Hons), MBChB, MRCP, MA (Clinical Education).

Dr Cathy Sherratt, Programme Leader, MA Clinical Education; Joint Chair, Postgraduate (Taught) Research Ethics Sub-committee. ORCID iD: https://orcid.org/0000-0002-5073-0023
