# Supplementary material for: Evaluation of the use of a handheld prescribing card in supporting Foundation year one doctors in End of Life (EOL) prescribing
Source: MedEdPublish (2016). 2019 Jul 24;8:157. [Version 1] doi: 10.15694/mep.2019.000157.1 (PMC10712460; doi:10.15694/mep.2019.000157.1)
Supplement: Peer review history

# Reviewer report: Reviewer response for version 1

**DOI:** 10.21956/mep.19879.r30063

**Published:** 2019-10-18

**Authors:** Ken Masters

**Affiliations:** 1 Sultan Qaboos University

This review has been migrated. The reviewer awarded 3 stars out of 5 An interesting paper in a very important area, and focusing on the evaluation of the use of a handheld prescribing card in supporting foundation year one doctors in End of Life (EOL) prescribing. The authors begin with a fairly detailed literature review indicating the lack of preparation among new doctors in the area of prescribing for End of Life care. They then detail the use of the card and its evaluation. Although the study is very small, it does provide some useful insights.Some areas that need to be addressed in the paper:• There is very little description of the setting, including the students’ background. This should be given in the Introduction, so that the reader is aware of the situational and educational context.• The software and version used in the data analysis needs to be indicated.• “There were almost equal numbers of males and females” The numbers should be given.• “Males favoured its use overall more frequently than females and the more mature doctors used the card more;” Numbers and statistical test results should be given. This is especially important because the sample is so small.• There are a few other places where descriptions are given and the number should be given instead.• “We noticed that there was a positive association with the introduction of the card and increased…” again, statistics are required.• It appears that some results (and even Methodology) are reported for the first time in the Discussion. Please move these to the appropriate sections.Minor:• “Quantitative data was analysed” should be “Quantitative data were analysed”• In the Results’ reporting, sentences should not begin with a number.So, overal,l an interesting paper on an important topic; some re-writing and re-structuring of the paper is, however, required.

Reviewer Expertise:

NA

No decision status is available

# Reviewer report: Reviewer response for version 1

**DOI:** 10.21956/mep.19879.r30064

**Published:** 2019-08-30

**Authors:** Barbara Jennings

**Affiliations:** 1 Norwich Medical School

This review has been migrated. The reviewer awarded 4 stars out of 5 The authors of this article have designed a tool (a prescribing card, see supplementary file 1) for postgraduate medical trainees, and undertaken an evaluation (see supplementary file 2 for the contents of the questionnaire) of its usefulness to foundation doctors working in end of life (EOL) care. In their literature review, they explore their case for this intervention, based on a lack of training about palliative care in undergraduate curricula coupled with reports of other successful projects that improved EOL care during early postgraduate training.The results section provided a comprehensive quantitative overview of the use of, and satisfaction with, the prescribing card - and explored motivating factors. In their discussion, the authors consider qualitative results too; from the comments about the format of the card, to fears about prescribing in EOL care.The data presented is only from a small number of participants (25 out of the 29 possible doctors completed the questionnaires). And I wondered if the prescribing card should have had version/date details, to ensure that up to date evidence-based practice is always adhered to – though I noted the link to the relevant online local guidelines. Moving from a hard copy to an online version could circumvent this problem. Do the authors plan to produce a web-based version that could be accessed from mobile devices in the near future? What would the advantages and potential barriers be for that intervention?Exploring recent published data on EOL prescribing errors would have also been useful as part of the literature review. Overall, I found this is a well written article, and the authors thoroughly explain their aims, intervention, and results analysis. The intervention and/or the literature discussed will be of interest to colleagues who are responsible for undergraduate and postgraduate training in palliative care.

Reviewer Expertise:

NA

No decision status is available
